# Thoracoscopic Esophageal Atresia with Tracheoesophageal Fistula Repair: The First Iranian Group Report, Passing the Learning Curve

**DOI:** 10.21699/jns.v5i3.344

**Published:** 2016-07-03

**Authors:** Mehran Hiradfar, Mohammad Gharavifard, Reza Shojaeian, Marjan Joodi, Reza Nazarzadeh, Alireza Sabzevari, Nazila Yal, Reza Eslami, Ahmad Mohammadipour, Ali Azadmand

**Affiliations:** 1 Department of Pediatric Surgery, School of Medicine, Mashhad University of Medical Sciences, Sarvar Children's Hospital, Endoscopic and Minimally Invasive Surgery Research Center, Mashhad, Iran; 2 Department of Pediatric Anesthesiology, School of Medicine, Mashhad University of Medical Sciences, Sarvar Children's Hospital, Endoscopic and Minimally Invasive Surgery Research Center, Mashhad, Iran

**Keywords:** Esophageal atresia, Tracheoesophageal fistula, Thoracoscopy, Neonate, Minimally invasive

## Abstract

**Background:** Thoracoscopic treatment of esophageal atresia and tracheoesophageal fistula (EA+TEF) is accepted as a superior technique at least in cosmetic point of view but it is considered as an advance endoscopic procedure that needs a learning curve to be performed perfectly. This is the first report of Iranian group pediatric surgeons in thoracoscopic approach to EA.

**Methods and Materials:** Since 2010, twenty four cases with EA+TEF underwent thoracoscopic approach in Sarvar Children Hospital (Mashhad -Iran). During the first 6 months, thoracoscopic approach to 6 cases of EA+TEF was converted to open procedure because of technical and instrumental problems. The first case of successful thoracoscopic EA repair was accomplished in 2010 and since then, 10 cases of EA+ TEF among 18 patients were treated successfully with thoracoscopic approach

**Results:** Overall conversion rate was 58.3% but conversion rate after the primary learning curve period, was 35.7%. The main conversion causes include difficulties in esophageal anastomosis, limited exposure and deteriorating the patient's condition. Anastomotic leak and stenosis were observed in 20% and 40% respectively. Overall mortality rate was 4.2%.

**Conclusion:** Thoracoscopic repair of esophageal atresia seems feasible and safe with considerable superiorities to the conventional method although acceptable results needs a prolonged learning curve and advanced endoscopic surgical skill. Clear judgment about the best surgical intervention for EA according to all cosmetic and functional outcomes needs further studies.

## INTRODUCTION

Treatment of esophageal atresia and trachea- esophageal fistula (EA+TEF) has been improved during last decades [1]. Acceptable outcome of EA repair is owed to several factors such as innovations in surgical technique and promotions in neonatal intensive care and neonatal anesthesia methods [2].

Since the development of minimally invasive interventions, these methods gradually became more popular in many fields and also in pediatric surgery [3].

Jacobaeus was the first one who describes the idea of thoracoscopy in 1912, using a cystoscope to release pleural adhesions [1]. Rothenberg reported the first advanced thoracoscopic repair of EA in 1999 and later on, thoracoscopic approach to EA with TEF came to practice [4]. Recently, thoracoscopic repair of EA is accepted as a superior technique at least in cosmetic point of view that eliminate a thoracotomy and avoid some complications such as a large scar, scoliosis and thoracic cage deformities and wing scapula.[5] during last 10 years, many academic centers reported their first experiences in thoracoscopic repair of EA+TEF and in a recent British survey, 46% of all pediatric surgeons choose thoracoscopic approach for EA Repair [6].

Thoracoscopic repair of esophageal atresia is considered as an advance endoscopic procedure and needs a learning curve to be performed perfectly [4]. Reports are still publishing, even from pioneer pediatric surgery centers to describe their new experiences in thoracoscopic EA+ TEF repair. This article is the first case series of Iranian group pediatric surgeons in thoracoscopic approach to EA.

## MATERIALS AND METHODS

Thoracoscopic approach for TE repair was tried in 2010 in Sarvar children Hospital (Mashhad University of Medical Sciences) for the first time in Iran. Since this time up to 2014, 24 cases (14 boys and 10 girls) with EA+TEF underwent thoracoscopic approach. Neonates with common type of EA with TEF were included if they hadn't major coexisting anomalies and the birth weight was more than 2000 grams.

Pros and cons of laparoscopic approach compare to open approach were explained in details for parents and possibility of thoracoscopic approach failure and risk of conversion was also discussed regarding the team experience and skill. We explained the benefits of MIS approach such as better cosmetic results, less pain and less functional sequels and morbidities if the procedure goes well. Finally we let them decide about treatment modality and inform consent was signed by the parents. 

Neonates were admitted in neonatal intensive care unit and preoperative assessments and preparations were performed. General anesthesia was induced and invasive monitoring of blood gases was maintained using pulse oximetry and capnogram. A 12F (white) Nelaton catheter was inserted to the proximal esophageal pouch through the mouth and an 8F (Blue) Nelaton catheter was inserted as a nasoesophageal tube.

The patient was positioned in the left lateral decubitus with 45 degree prone tilt at the left edge of the operation table with a small pad under the chest. Pelvic was fixed to the table by a strip band and the right arm was placed over the head. Mild reverse Trendelenburg position with left tilt was obtained. Surgical team positioning is shown in figure 1. 

**Figure:**
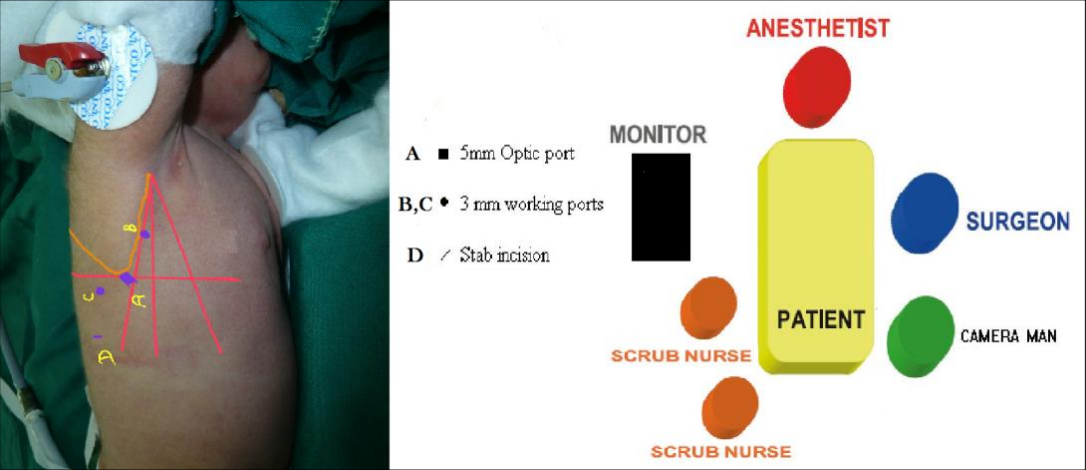
Figure 1: Operative team and instruments and patient positions.

All operations were performed by senior surgeon of department who had been passed preliminary courses (first author) and other surgeons were contributing as assistants. Operation was begun with insertion of a 5mm port at posterior auxiliary line just near to the tip of scapula. After checking proper position of the first port in plural space, gas insufflation was started with the flow of 4 lit/min maintaining a 5mmHg intra pleural pressure. Transient hypoxemia and hypercarbia usually occurred at the beginning due to the lung collapse and pressure effect on mediastinal components. Anesthesiologist had a 20 min time period to readjust the ventilation parameters and stabilize the patient. This relaxing interval let the right lung to collapse partially against the positive plural pressure. In order to shorten this lag time, Authors preferred to insert an atraumatic locking grasper in to the plural space through a small stab incision in the 7th intercostal space at the mid-axillary line in order to grasp the upper lobe of the right lung and push it down to provide a better work space. Then two 3mm ports were inserted just 20-30mm away from the camera port along the anterior rim of scapula in a triangular fusion.(Fig. 1) Parietal pleura was dissected by cautery to expose Azygos arch and proximal and distal parts of esophagus. Azygos vein transected just in the case of limited exposure otherwise, exploration of TEF was performed bellow the Azygos arch and ligated and dissected. Proximal esophageal pouch was detected while pushing down the 12Fr oral tube as a guide. After full mobilization of the proximal pouch, esophageal anastomosis was accomplished over an 8F nasogastric tube by 5-0 Vicryl. At the end of our learning curve we introduced some technical innovation like tube handling anastomosis which was performed in the last 5 cases to facilitate suturing. In this modification we passed an eight French catheter through the anastomosis in to the distal part after the first stiches on the antro lateral esophageal wall. We grasp this catheter for better and less traumatic tissue handling and better exposure of posterolateral wall to complete the anastomosis.

Anastomotic site was covered by native fibrin glue in some cases (Fig. 2).

**Figure:**
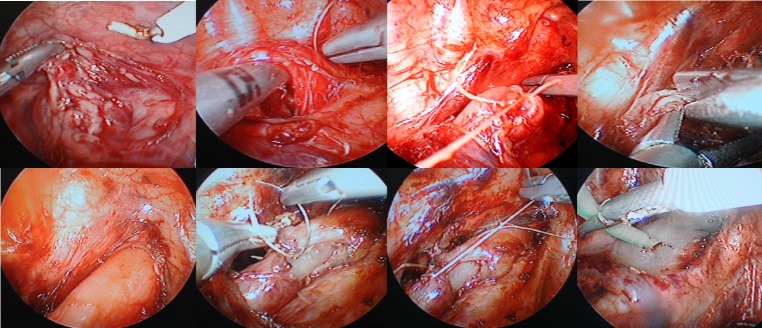
Figure 2: Different Stages of thoracoscopic TEF ligation and EA repair.

The operation was converted to open approach if any deterioration in patient hemodynamic status was happened or if the operation prolonged for more than 4 hours or any stage of operation remained without progress for an hour. Chest tube was inserted and the patient was transferred to the NICU under ventilation support. Nasogastric tube was withdrawn in the third to fifth postoperative day if the postoperative course was uneventful. Oral feeding was started gradually and chest drain was monitored for any sign of anastomotic leakage. Esophageal barium study was performed in doubt of anastomotic leakage or oral diet intolerance.

## RESULTS

Since 2010, 24 cases of EA+ TEF underwent thoracoscopy in Sarvar Pediatric Hospital (Mashhad-Iran). During the first 6 months, surgical approach to 6 patients with EA+ TEF was initiated with thoracoscopy but the operation was converted to open procedure because of technical and instrumental problems. The learning curve is shown in table 1. 

**Figure:**
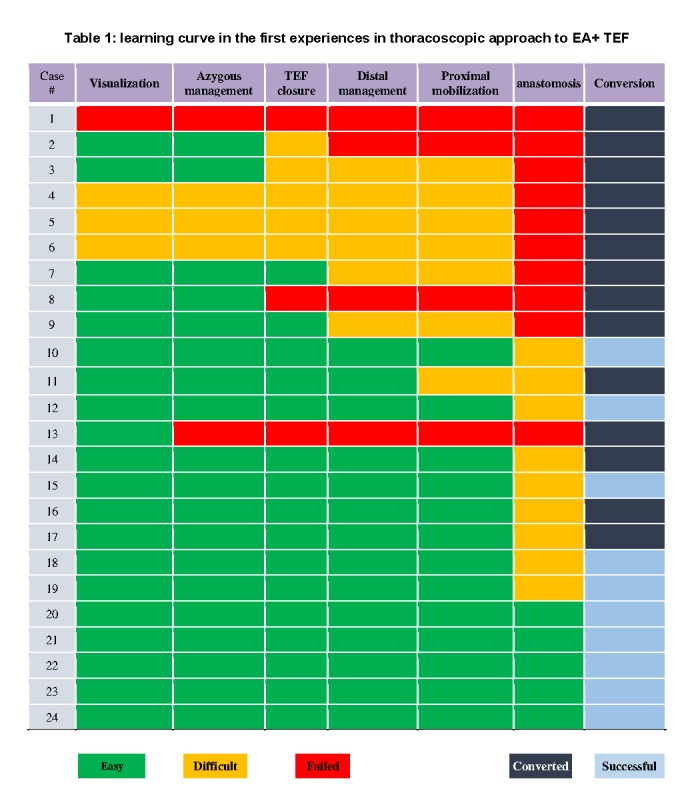
Table 1: learning curve in the first experiences in thoracoscopic approach to EA+ TEF

The first case of successful thoracoscopic EA repair was accomplished in 2010 and since then, 10 cases of EA+ TEF have been treated successfully with thoracoscopic approach. Mean age among these neonates was 2.1±1.6 days. Weight average was 2845±861 grams. Overall conversion rate was 58.3% but conversion rate after the first 10 cases of learning curve period reduced to 35.7%.

The main conversion causes were difficulties in esophageal anastomosis and knotting in small work space (6 cases), limited exposure due to right aortic arch (2 patients) and deteriorating patient's condition and hypoxia (2 cases), prolonged operation time (2 cases) bleeding from Azygos vein (one case) and inappropriate instruments (one case). 

Among patients with successful thoracoscopic approach, the average operative time was 220±76.4 minutes. Median neonatal intensive care admission time was 8.9±15.3 days and median time to discharge from hospital was 16±15.9 days. 

Mean time to start oral feeding was 4.6±3.35 days and barium study wasn't done routinely. Anastomotic leak was observed in 2 cases among those with successful thoracoscopic repair (20%) and 2 cases among open approach (14.3%) all cases were managed by conservative approach. Esophageal stenosis was detected in 4 patients (40%) among those with successful thoracoscopic repair and 5 (35.7%) cases among open approach in postoperative course that were all managed by dilatation. Endoscopic dilatation ranged from 1 to 4 times in these cases. Recurrent fistula wasn't seen in our patients.

Mortality was reported in one case among all 24 patients that was premature baby and converted to open approach because of difficulties in knotting (among primary six cases in the learning curve). This patient died due to respiratory failure and mechanical ventilation dependency and sepsis in the early postoperative course in intensive care unit. So overall mortality rate was 4.2% and mortality rate was zero among patients with successful thoracoscopic approach.

## DISCUSSION

Thoracoscopic repair of esophageal atresia is considered as an advanced endoscopic procedure that needs a high skill in minimally invasive surgery and a perfect team working between surgical group , assistants and anesthesiologist during operation and also neonatal intensivist and pediatric gastroenterologist and cooperative parents in the postoperative course.[7]

Thoracoscopic esophageal atresia repair like other advanced surgical skills needs a learning curve to gain acceptable results. In the first 6 months of our experiences in Thoracoscopic EA repair, we just tried to improve our technique, found out the pitfalls and applied the best arrangement for the operation team and improved the instruments so each operation was a step forward in practicing minimally invasive (MIS) approach to EA repair. Considering the patient safety during the learning curve (First six months) our time threshold for conversion to open procedure was as short as just two hours. This may be the main reason for frequent conversions to open approach in the primary patients. During the second six months of our practice we readjusted the time threshold to 3 hours; we converted the MIS approach to conventional method when any stage of operation remained without progress for an hour. 

Among our last 10 patients, 8 successful thoracoscopic approaches were accomplished so the conversion rate was acceptable (20%). Previously published articles reported a conversion rate of 3%-30% in different centers.[4,8,9]

The eighth patient among our cases was converted to thoracotomy due to bleeding from Azygos while cutting the vein after ligation. This accident encouraged us to leave the Azygos intact unless it interfered with exposure. We succeeded to expose the TEF below the Azygos arch easily in almost all cases but anastomosis was accomplished in front of the Azygos vein.

Anastomotic stricture rate after thoracoscopic EA+TEF repair ranged from 15% to 45% in different published articles.[10] Although our anastomotic leak and stenosis were higher than recently published articles but all these complications were managed by conservative approach and dilatations. In a large series of thoracoscopic EA repair during last 10 years on 72 cases, postoperative leak and stenosis in the first 5 years of their experience were 22% and 38% respectively that reached to 8% and 19% in the last 5 years.[4]

Other risk factors for anastomotic leak and stenosis include gastroesophageal reflux, ischemia and excessive tissue manipulations and injuries. In order to facilitate the anastomosis with minimal tissue manipulations, we have introduced a technical innovation in our last 5 cases named as "tube handling anastomosis" that decreased tension and provide better exposure by hanging the distal esophagus to the esophageal tube [11]

One of the most complicated sequences of thoracoscopic EA+TEF repair is the esophageal anastomosis and suturing, tying and knotting in a small work space. This may cause a primary hesitation among many pediatric surgeons to decide minimally invasive approach to EA. In a recent British survey, only 46% of pediatric surgeons were interested in starting thoracoscopic approach for EA [6], but proven advantages of MIS approach in EA repair such as eliminating a thoracotomy and other related complications including thoracic deformities, rib fusion and scoliosis, shoulder fixation and elevation, winged scapula and breast and muscular mal-developments etc. [12-16] indicate the superiority of MIS approach to the conventional methods.

Several innovations and experimental studies have been published that aimed to introduce a method to facilitate esophageal reconstruction [3, 17] and perhaps this stage of operation would be facilitate by surgical and instrumental advances in future.

Comparing the outcome and functional evaluations between open and thoracoscopic approach, recent meta-analysis didn't show significant difference [18]. Considering the clear cosmetic and anatomical benefits of MIS approach in EA+TEF, all these complexities and prolonged learning curve shouldn't disappoint pediatric surgeons to develop MIS approaches especially in neonatal surgery.

## CONCLUSION

Thoracoscopic repair of esophageal atresia seems feasible and safe with considerable superiorities to the conventional method although acceptable results needs a prolonged learning curve and advanced endoscopic surgical skill. Clear judgment about the best surgical intervention for EA according to all cosmetic and functional outcomes needs further studies.

## Footnotes

**Source of Support:** Endoscopic and minimally invasive surgery research center of Mashhad University of medical sciences

**Conflict of Interest:** None
